# Building a strong European alliance for personality disorder research and intervention

**DOI:** 10.1186/s40479-018-0082-z

**Published:** 2018-04-02

**Authors:** Lars Mehlum, Anthony Bateman, Henk Jan Dalewijk, Stephan Doering, Andres Kaera, Paul Anthony Moran, Babette Renneberg, Joaquim Soler Ribaudi, Sebastian Simonsen, Theresa Wilberg, Martin Bohus

**Affiliations:** 1 National Centre for Suicide Research and Prevention, Institute of Clinical Medicine, University of Oslo, Oslo, Norway; 2 University College, London, University of Copenhagen, London, UK; 3 Anna Freud National Centre for Children and Families, London, UK; 4 Private practice, Weesp, The Netherlands; 5 Department of Psychoanalysis and Psychotherapy, Medical University of Vienna, Vienna, Austria; 6 Kanta-Häme Central Hospital, Hämeenlinna, Finland; 7 Centre for Academic Mental Health, Department of Population Health Sciences, Bristol Medical School, University of Bristol, Bristol, UK; 8 Freie Universität, Berlin, Germany; 9 Universidad Autonoma de Barcelona, Barcelona, Spain; 10 Stolpegaard Psychotherapy Centre, Copenhagen, Denmark; 11 Department of Research and Development, Division of Mental Health and Addiction, Oslo University Hospital and Institute of Clinical Medicine, University of Oslo, Oslo, Norway; 12 Institute of Psychiatric and Psychosomatic Psychotherapy; Central Institute of Mental Health, Mannheim, Heidelberg University, Mannheim, Germany

**Keywords:** Personality disorder, Dissemination, Network, Non-governmental organization

## Abstract

People with personality disorders frequently face stigma, ignorance and pessimism regarding the treatability of their disorders. This is despite substantial progress that has been made in developing a number of effective evidence based psychotherapeutic treatments. However, expertise in how to systematically deliver these treatments in a sustainable way throughout Europe is largely lacking. To bridge the gap between evidence based treatments and their implementation in health services, the European Society for the Study of Personality Disorders is currently building a new alliance of experts to promote personality disorder scholarship, and to support the development of clinical expertise and systematic treatment implementation throughout Europe. The aim of this paper is to describe how the Society is currently using its interdisciplinary and international roster of experts to address the specific treatment and research needs of the European personality disorder field, particularly to countries in which expertise in the field is less developed.

## Introductory note from the joint Editors-in-Chief

We are pleased to present the following Guest Editorial entitled “Building a Strong European Alliance for Personality Disorder Research and Intervention” by Lars Mehlum and colleagues. BPDED is supported by affiliations with several professional societies, one of which is the European Society for the Study of Personality Disorders (ESSPD), a growing and influential organization that encourages scholarship, education, research, and treatment for patients with personality disorders. Funding for all of these priorities is woefully inadequate throughout Europe and, indeed, throughout the rest of the world. The Guest Editorial below is co-authored by a roster of leading experts in Europe who are dedicated to addressing these needs. The efforts and goals of these thought leaders are detailed below, and we encourage readers to review this information and to participate in this campaign for progress to improve the lives of patients with personality disorders.

## Background

Personality disorders are highly prevalent both in the general [1] and in clinical populations [2] and they are strongly associated with poor social adjustment [3], high prevalence of psychiatric [4] and somatic [5] comorbidity, reduced quality of life [6] and increased mortality [7]. These disorders emerge in adolescence [8], and they regularly lead to high use of health services. Consequently, personality disorders pose a strong economic burden to society, the magnitude of which is larger than for several other highly prevalent mental disorders such as major depression or generalized anxiety disorder [9]. There are therefore strong arguments for strengthening systems for early detection and diagnosis of personality disorders and for offering people with personality disorders early intervention as well as effective treatment. Regrettably, this is currently hardly the case anywhere in the world despite the substantial increase in our knowledge about effective treatments [10]. There is a huge gap between the real needs for personality disorder specific treatments in the population and the treatment that is being offered.

European governments have jointly acknowledged that there is a strong need for improvement in the quality of treatments and access to treatments for people with mental health problems [11]. Building more platforms for international and regional sharing of experience, dissemination of knowledge on best practice, and helping create a more competent workforce are among initiatives recommended by European governments in order to modernize mental health care and make it more accessible to all the peoples of Europe. This challenge is huge and no single country, organization or professional group can hope to overcome it alone. With respect to building better services for people with personality disorders, it is, however, our strong belief that researchers and clinicians expert in personality disorders have a major role to play in helping politicians’ visions become reality through more concerted action. In this paper we will describe how we are currently building what we hope will grow into a strong European alliance for personality disorder research and intervention to support the proliferation of better evidence based treatment services for the peoples on the continent.

## The origin of the European Society for the Study of personality disorders (ESSPD)

The ESSPD was formed in 2010 with the aim of promoting interest throughout Europe about all aspects of personality disorder. At the time there was no clear organisation promoting the field of personality disorder within European health systems. The European region of the International Society for the Study of Personality Disorder (ISSPD) supported international interaction at conferences, but there was no specific organisation either focusing on strategies for future development in the field of personality disorder in Europe or co-ordinating the research and clinical activities of European professionals. The founding of the society arose out of these concerns with recognition that there was inadequate development of research into personality disorders within Europe and limited interest from European governments and mental health services in implementing evidence based treatment services for people with a personality disorder.

Upon an initiative of Dr. Thomas Rinne of the Netherlands a new European Society for the Study of Personality Disorders (ESSPD) was established with him as the founding president and with the additional support of Prof Martin Bohus from Germany, Prof Anthony Bateman from the UK, and Drs Henk-Jan Dalewijk and Ad Kaasenbrood, both from the Netherlands. At the first International conference on borderline personality disorder (BPD) organized by Martin Bohus in Berlin in 2010 under the auspices of the new society, the first constitutional general members’ meeting of the ESSPD took place. During this meeting an international group of mental health professionals agreed to develop proposals for the policy of the society focusing on personality disorder in Europe and the first board of directors was elected with Anthony Bateman as the first elected president. Due to the great success of this first international BPD congress (more than 1400 participants), one of the first decisions of the newly founded ESSPD was to plan for international BPD congresses on a biennial basis. Successful international congresses have now been organised in Amsterdam (2012), Rome (2014) and Vienna (2016) and future congresses are planned for Barcelona / Sitges in 2018 and Antwerp in 2020.

## ESSPD’s vision and mission

The ESSPD has already been very successful in organizing congresses to which large numbers of experts from all major walks of personality disorder research and practice have chosen to come together. The vision of the ESSPD is to form a unique interdisciplinary and international network of all those experts who would like to join forces to help build a more competent mental health workforce needed to modernize care services and make them more accessible to all the peoples of Europe. The ESSPD realizes that this vision cannot rely on a loose network alone. In order to increase its effectiveness and impact the society is therefore inviting experts in personality disorder research, clinical practice, training and/or policymaking throughout Europe to join as members of what we see as no less than *an academy of excellence*. In the following we will describe some of the objectives members of this academy are currently working to achieve.

## Disseminating knowledge in personality disorder treatment and intervention across Europe

Across Europe, mental health professionals have highly variable knowledge on how to diagnose and treat personality disorders. The variability ranges from the situation in some countries where personality disorder diagnoses are rarely made to the situation in others where state of the art national guidance is available along with quality indicators for treatment. As a measure to help improve the situation in countries with a high need, the ESSPD has started organizing workshop conferences where trainings in practical skills of evidence-based treatments for personality disorders are delivered thus building clinicians’ competencies. These conferences have a fixed format with a limited number of plenary speakers who also each provide a half-day workshop twice. We strive for theoretical heterogeneity with no model being dominant in the structure of the meeting. For example, the first workshop-conference organized in Tallinn, Estonia in 2015 gave participants the possibility of getting acquainted with Schema Therapy through Arnoud Arntz, Mentalization Based Therapy for Antisocial personality disorder through Anthony Bateman, Dialectical Behaviour Therapy for Posttraumatic Stress Disorder through Martin Bohus, Metacognitive Interpersonal Therapy for Avoidant personality disorder through Giancarlo Dimaggio, Social Psychiatric Management through Ad Kaasenbrood, and Cognitive Behavioural Therapy for Avoidant personality disorder through Babette Renneberg. This conference was well attended with 46% of participants coming from Estonia and another 35% from neighbouring Baltic countries and Finland. The conference was a success, with 83% of participants surveyed after the conference estimating that the conference would change their practice. Since this first workshop-conference more similar events have been implemented. The feedback from participants consistently indicates that there is great need for disseminating knowledge about evidence based and effective methods of treatment for patients with personality disorders. The ESSPD is consequently continuing to organize workshop-conferences primarily in Eastern-European countries where treatments are generally less developed. Affordable and accessible distribution of knowledge and therapeutic skills is a key aim of the ESSPD and these meeting contribute to fulfilling that aim. The next workshop conference will be held in Budapest, Hungary in 2019.

## Training young researchers

It is not only clinical competence in personality disorder specific treatments which are unevenly distributed in Europe. The same is the case with research, where large regions have little or no activities. The ESSPD, therefore, aims to promote recruitment of and support to young researchers specializing in personality disorder studies from all over Europe, and particularly to help build research networks and resources in Eastern European countries. We are convinced that developing and disseminating knowledge based treatments in the years to come must be guided by research and this will, in turn, strongly depend on the development of young researchers and researcher-clinicians specializing in personality disorder studies. The ESSPD is currently preparing a summer school programme to which young researchers from Europe will be invited to apply. Accepted candidates will present and discuss their projects in the realm of personality disorders research with a faculty of distinguished senior experts in the field. The participation fee will be kept low through subsidies from the ESSPD budget and scholarships will be made available to young researchers from Eastern European countries.

We are currently also building a database of competencies where all major groups of personality disorder researchers in Europe will be found with their special areas of expertise and research projects. We hope that this resource will help young researchers more easily identify senior scientists and their groups and vice versa help senior researchers to connect with talented junior researchers.

The ESSPD workshop conferences are valuable venues for junior clinicians and researchers interested in personality disorders. Similarly the ESSPD is using the large biennial international conferences to attract and support junior researchers. The society has established awards for junior researchers and these awards are linked to a fellowship that can be spent for a research project or a visit to a renowned research group. Scholarships are also made available to facilitate the participation of young researchers who wish to present their research at the ESSPD congresses.

## Recruiting and engaging experts in personality disorder research, clinics, training and policymaking

The sustainability and success of most of the initiatives mentioned above will depend heavily on more European experts in personality disorder research and clinical practice joining forces to reach more colleagues in more countries. This is why the ESSPD is eager to recruit more members with a high level of expertise in research, clinical work, training and/or policymaking. In line with the idea of transitioning into an academy of excellence, plans were made to include more outstanding specialists in the field, thus also increasing membership numbers and adding influence to the organization.

Not surprisingly, also in the roster of ESSPD there has been a clearly uneven distribution of members; historically, the Netherlands, UK and Italy together accounted for almost half (46%) of the members. A number of steps have therefore been taken to expand the number of members and particularly make the geographical distribution more even. These initiatives have yielded an increase of about 40% in overall members’ numbers. In some countries – Germany, Norway, Denmark and Spain, for example - the numbers have at least tripled, becoming more in line with the amount of research on personality disorders these countries are making. A closer look at what category of experts our new members come from shows that 88% were researchers, 81% clinicians, 10% policy makers, 57% trainers and 7% organizers. Obviously, the majority of these members have competencies in several areas.

In our on-going recruitment efforts we also concentrate on countries, such as France, Switzerland, Belgium and Sweden, where we know there are significant numbers of researchers contributing to the personality disorder literature, but from which few have so far become ESSPD members.

To support networking and communication among the growing number of members, the ESSPD is now developing a user-friendly web platform at the www.esspd.eu. As part of the recruitment process, new members are asked to complete a form specifying information about their fields of interest as well as on-going research projects. Members will also be asked for their consent for this information to be posted on a restricted members’ only area of the ESSPD website. Overall, we envision that, with time, this platform will be used to promote more collaboration, debate and sharing between experts across Europe and to strengthen the field of personality disorder research.

The goal of building an *academy of excellence* in personality disorder research, clinical practice, training and policy making is ultimately to help build a mental health work force in Europe that can better deliver high quality services to those in need in all countries. We believe that all European countries have large challenges in this respect. But we also believe that huge savings in terms of time, money and human resources can be made when experts across the continent work more systematically together. For example interdisciplinary and international teams of experts can jointly create updated evidence based clinical guidelines, both at the European level and in each country. A co-ordinated group of experts can also much more effectively impact on important issues such as revisions of diagnostic systems. The current process with a new revision of the international classification of diseases (ICD-11) has shown how important it is that researchers and clinicians collaborate and join forces to make sure that those who are deciding upon the revisions get the best possible advice [12].

## Conclusions

Personality disorders may be effectively treated through several evidence based psychotherapeutic interventions developed over recent decades [13]. This gives hope to the many in need of treatment. However, since expertise in systematically delivering these treatments in a sustainable way is largely lacking, huge efforts are needed to support such delivery and implementation in Europe. At the same time, more research is needed to better delineate what are the mechanisms of change during effective treatment and what are the essential components of treatments. The vision and mission of the ESSPD is to help overcome some of these challenges by building a strong alliance of experts to promote personality disorder research and intervention throughout Europe.
